# Holiday ratio of hospitalization and 30‐day readmission rates among cancer patients after major surgery

**DOI:** 10.1002/cam4.4482

**Published:** 2021-12-14

**Authors:** Ling‐Jan Chiou, Hsiu‐Min Chen, Li‐Fei Pan, Ching‐Chih Lee

**Affiliations:** ^1^ Department of Health Business Administration Department of Nursing, and Department of Oral Hygiene Meiho University Pingtung Taiwan; ^2^ Department of Medical Education and Research Kaohsiung Veterans General Hospital Kaohsiung Taiwan; ^3^ Department of Medical Affair Administration Kaohsiung Veterans General Hospital Kaohsiung Taiwan; ^4^ Department of Otolaryngology, Head and Neck Surgery Kaohsiung Veterans General Hospital Kaohsiung Taiwan; ^5^ Institute of Hospital and Health Care Administration National Yang Ming Chao Tung University Taipei Taiwan; ^6^ School of Medicine National Defense Medical Center Taipei Taiwan

**Keywords:** cancer management, colorectal cancer, prognosis, surgery

## Abstract

**Background:**

To determine the association of 30‐day readmission with weekend discharge and the number of holiday days during a hospital stay (holiday ratio).

**Methods:**

This retrospective cohort study used the clinical research database and cancer registry data of our hospital from January 1, 2011 to December 31, 2017. Patient characteristics, tumor factors, clinical laboratory data, and proxies of continuity of care, such as weekend discharge or holiday ratio (holiday days/total hospitalization days), received statistical analysis. Multivariate logistic regression identified the independent factors for 30‐day potentially avoidable readmission rate (PAR).

**Results:**

Of 1433 patients receiving tumor resection, 520 (36.29%) had colon cancer; 440 (30.70%) had head and neck cancer (HNC), and 473 (33.01%) had other cancers (lung, liver, and prostate). The rate of 30‐day PAR was 6.3% for those with colon cancer, 8.6% for HNC, and 3.6% for other cancers. The 30‐day PAR did not significantly differ by discharge on a weekend versus weekday for those with colon cancer (8.33% vs. 5.90%; *p *= 0.379), HNC (7.06% vs. 9.01%; *p *= 0.566), or other cancers (0.00% vs. 4.28%; *p *= 0.960). Colon cancer patients with holiday ratio >0.3 had a higher readmission rate (9.58% vs. 4.82%, *p *= 0.041). In multivariate analysis, a holiday ratio >0.3 (adjusted odds ratio 2.16; 95% Confidence Interval, 1.05–4.39) in those with colon cancer was an independent predictor of 30‐day PAR.

**Conclusions:**

Weekend discharge after major surgery did not affect 30‐day readmission rates in cancer patients, but the holiday ratio did affect 30‐day PAR for those with colon cancer.

## Graphical abstract

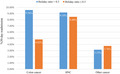

**Lay summary**

The continuity of care on weekend discharge and the number of holiday days during a hospital stay was the important factor for 30‐day hospital readmissions among cancer patients after major surgery. Among patients hospitalized in a medical center, a higher holiday ratio during hospitalization in colon cancer patients affected the likelihood of subsequent 30‐day hospitalization. Improving the hospitalist scheduling and appropriate coordination of care protocols on weekends during hospitalization can reduce the subsequent 30‐day readmission and mortality rates in colon cancer patients.

## 1 INTRODUCTION

In the United States, the aggregate hospital costs of hospital readmissions for four high‐volume diseases reached $7 billion in 2013.^1^, ^2^ Of these, 18% were 30‐day readmissions, and most readmissions were not scheduled.^3^ The three strategies most used to reduce readmissions are: identifying high‐risk groups, providing greater continuity of care, and increasing patient education.^4^ Besides identifying high‐risk patients, the most critical step to reducing hospital readmission rates, and the associated higher costs and higher rates of short‐term complications, is to improve continuity of care.^5^, ^6^ Continuity of care consists of (1) interpersonal continuity, such as care provided by the same central providers; (2) longitudinal continuity, as in discharge planning; (3) management continuity, as in shared collaborative care; and (4) informational continuity, as in the use of shared records.^7^, ^8^ Previous studies have used several proxies to measure continuity of care, such as a continuity of care index or an outpatient visit within 7 days of discharge.^9^, ^10^, ^11^ Indexes for continuity of care include the Continuity of Care Index, usual provider index (UPC), or Sequential Continuity Index.^9^, ^12^ However, these might reflect only the interpersonal continuity, and have been most commonly used in the outpatient context.

Suitable indices to represent continuity of care during hospitalization are scant. The main measure of continuity of care was derived from interpersonal continuity, measuring the degree of cross‐over coverage. Cross‐over coverage in the hospital means that the patient receives care from more than one physician, which could result in decreased continuity of care and has been associated with poor outcomes in turn.^13^ Previous studies revealed that the weekend UPC, identified through the percentage of clinical notes provided by the original physician during a hospitalization, was associated with length of stay, but neither the readmission rate nor mortality.^14^ Hospitalist schedule arrangement could also be a proxy of continuity of care for inpatients. High continuity of hospitalist schedules was associated with lower 30‐day mortality and readmission rates.^15^ Several studies have also treated discharge on a weekend or holiday as indicating low continuity of care.^16^, ^17^ However, there is no widely accepted method at present to measure continuity of care.

The aim of this study was to develop a new and simple proxy of continuity of care, which could enable a strategy to improve healthcare quality in a referral medical center. Besides above‐mentioned indices, we also created a new indicator, holiday ratio. We hypothesized that the continuity of care was disrupted by the insufficient staff during holidays or weekends. Patients with similar length of stay might experience different holidays or weekends due to different admission date (Figure S1). Admission with higher holiday ratio could incur worse outcomes, such as readmission or further complications.

## 2 MATERIALS AND METHODS

### 2.1 Patient demographics and database

Data were obtained from our hospital Cancer Registry database and Clinical Research database, on adult patients with newly diagnosed cancer who had undergone primary tumor resection between January 1, 2011 and December 31, 2017. We selected patients with the most prevalent types of solid tumors and divided the cohorts into three groups: colon cancer; head and neck cancer (HNC); and lung, liver, and prostate cancer (other cancers).

We excluded the data of patients without complete clinical data or patients with distant metastasis at diagnosis. The registry database included patients’ demographic data such as age, gender, length of hospital stay, tumor site, and Charlson Comorbidity Index score (CCIS).^18^ We also extracted clinical data such as hemoglobin level, sodium level, and white blood cell count (WBC). P staging was performed according to the cancer staging recommended by the American Joint Committee on Cancer (7th edition).^19^

The dependent variable was the 30‐day potentially avoidable readmission rate (30‐day PAR) after discharge among patients receiving primary tumor resection. We used the SQLape algorithm to identify readmissions for unavoidable reasons such as scheduled chemotherapy, radiotherapy, follow‐up or rehabilitation treatment, or specific surgical procedures; these were not recorded as events.^20^

We tried to find a new proxy which could represent continuity of care during hospitalization. The most commonly used was discharge on a weekend.^6^, ^16^, ^21^, ^22^, ^23^, ^24^, ^25^ According to the literatures, patients discharged on the weekends or holidays may have low continuity of care because of personnel staffing levels, procedures delay, and loss of information on handoffs … et al. We also created a new indicator, named holiday ratio, determined as:

$$\mathrm{Holiday}\;\mathrm{ratio}=\frac{\mathrm{Holiday}\;\mathrm{days}}{\mathrm{Total}\;\mathrm{length}\;\mathrm{of}\;\mathrm{stay}}$$

The cutoff point for holiday ratio was 0.3. Patients with a holiday ratio >0.3 were defined as having low continuity of care (Figure S2 and Table S1).

### 2.2 Statistical analysis

SAS version 9.4 (SAS, Inc.) was used to analyze the data. Categorical variables, such as age stage, gender, cancer stage, discharge day, holiday ratio, CCIS, and other factors were analyzed with Pearson's chi‐square or Fisher's exact test. Continuous variables, such as age and length of stay, were compared with one‐way analysis of variance. Univariate and multivariable logistic regression models were used to estimate the odds of 30‐day PAR. The gender, age group, and significant variables with a *p *≤ 0.1 in univariate models were included in the multivariate analysis. Backwards stepwise logistic regression was used to determine the variables in our final model. A two‐side *p* value of <0.05 was considered as statistically significant. Another goal of this study was to exam the relationship the accumulation of the deficits survival between with and without 30‐day readmission. A Kaplan–Meier curve depicted this relation category for the colon cancer HNC and other cancer patients.

## 3 RESULTS

Of the 1433 discharged patients, the mean age was 63.6 ± 13.3 years, and 74.4% of all patients were men. The samples consisted of 520 (36.3%) patients with colon cancer, 440 (30.7%) with HNC, and 473 (33.0%; lung 185, liver 221, and prostate 67) with other cancers. Differences in age, gender, length of stay, discharge day, P stage, CCIS, hemoglobin level, sodium level, WBC, and 30‐day PAR of the patients in the three groups are listed in Table 1.

**TABLE 1:** Cohort demographics and clinical characteristics

Variables	Colon cancer	HNC	Other cancer^b^ (lung, liver, and prostate)	
	*n* = 520	*n* = 440	*n* = 473	*p* value
Age (mean ± SD)	69.1 ± 13.4	56.2 ± 11.3	64.4 ± 11.5	<0.001
Age				
≥65 years	327 (62.9)	90 (20.5)	249 (52.6)	<0.001
<65 years	193 (37.1)	350 (79.5)	224 (47.4)	
Sex				
Male	322 (61.9)	411 (93.4)	333 (70.4)	<0.001
Female	198 (38.1)	29 (6.6)	140 (29.6)	
Length of stay (mean ± SD)	25.4 ± 16.0	24.2 ± 11.9	23.2 ± 11.7	0.036
Discharge day				
Weekday	424 (81.5)	355 (80.7)	397 (83.9)	0.408
Weekend day	96 (18.5)	85 (19.3)	76 (16.1)	
Pathological stage				
I + II	248 (47.7)	140 (31.8)	363 (76.7)	<0.001
III + IV	272 (52.3)	300 (68.2)	110 (23.3)	
CCIS				
0	23 (4.4)	370 (84.1)	101 (21.4)	<0.001
1–2	264 (50.8)	55 (12.5)	192 (40.6)	
≥3	233 (44.8)	15 (3.4)	180 (38.1)	
Hemoglobin				
≥12 (g/dl)	148 (28.5)	218 (49.5)	182 (38.5)	<0.001
<12 (g/dl)	372 (71.5)	222 (50.5)	291 (61.5)	
Sodium				
≥135 (mEq/L)	421 (81.0)	367 (83.4)	405 (85.6)	0.144
<135 (mEq/L)	99 (19.0)	73 (16.6)	68 (14.4)	
WBC				
≤10,000 (/mm^3^)	321 (61.7)	273 (62.0)	270 (57.1)	0.218
>10,000 (/mm^3^)	199 (38.3)	167 (38.0)	203 (42.9)	
30‐day PAR^a^				
Yes	33 (6.3)	38 (8.6)	17 (3.6)	0.006
No	487 (93.7)	402 (91.4)	456 (96.4)	

Abbreviations: CCIS, Charlson Comorbidity Index score; HNC, head and neck cancer; SD, standard deviation.

^a^ 30‐day PAR: 30‐day potentially avoidable readmission.

^b^ Other cancer *n* = 473 (including 185 of lung, 221 of liver, 67 of prostate cancer).

**FIGURE 1:**
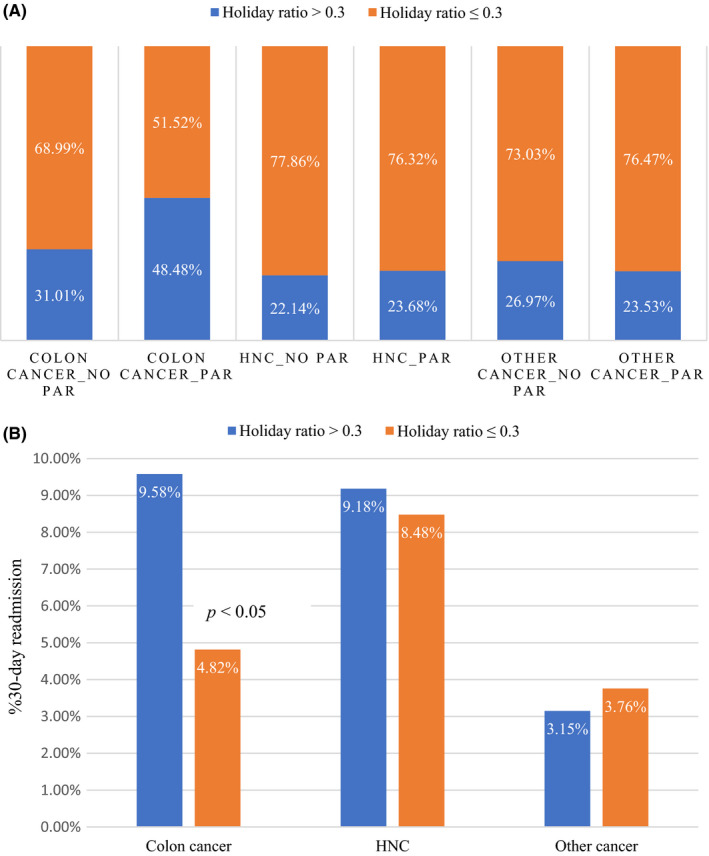
(A) Distribution of Holiday ratio >0.3 and ≤0.3. (B) 30‐day readmission rate for colon cancer, HNC (head and neck cancer), and other cancer based on Holiday ratio. *p *< 0.05 for Holiday ratio in colon cancer

Figure 1 shows analysis of the three groups by 30‐day PAR. Figure 1A shows that the percentage of patients with colon cancer with a holiday ratio >0.3 who had a 30‐day PAR was 48.48%, a rate much higher than that in other groups. Table 2 shows the association of baseline characteristics with the 30‐day PAR of the different groups. The rate (9.58%) of 30‐day PAR in colon cancer patients with a holiday ratio >0.3 was the highest among all groups and significantly different from that of patients with colon cancer whose holiday ratio was ≤0.3 (4.82%). Patients with colon cancer who were readmitted within 30 days were more likely to have a holiday ratio >0.3 (*p *= 0.041) and be older (*p *= 0.017). Patients with HNC who were readmitted within 30 days were more likely to have advanced pathological stage disease (*p *= 0.014), CCIS 1–2 versus CCIS 0 (*p *= 0.010), sodium <135 milliequivalents per liter (*p *= 0.012), and leukocytosis (*p *= 0.010). There were no significant predictors for 30‐day PAR among those with other cancers. Figure 1B shows the 30‐day PAR rate in three subgroups based on holiday ratio.

**TABLE 2:** Association between baseline characteristics and 30‐day PAR of patients

	Colon cancer				HNC						Other cancer (lung, liver, and prostate)			
Variables	Without 30‐day PAR^b^	With 30‐day PAR	Crude OR (95% CI)^c^	*p* value		Without 30‐day PAR	With 30‐day PAR	Crude OR (95% CI)	*p* value	Without 30‐day PAR		With 30‐day PAR	Crude OR (95% CI)	*p* value
Discharge day														
Week day	399 (81.9)	25 (75.8)	Ref.			323 (80.3)	32 (84.2)	Ref.		380 (83.3)		17 (100.0)	Ref.	
Weekend day	88 (18.1)	8 (24.2)	1.45 (0.63–3.32)	0.379		79 (19.7)	6 (15.8)	0.77 (0.31–1.90)	0.566	76 (16.7)		0 (0.0)	<0.001 (<0.001–>999)	0.960
Holiday ratio^a^														
≤0.3	336 (69.0)	17 (51.5)	Ref.			313 (77.9)	29 (76.3)	Ref.		333 (73.0)		13 (76.5)		Ref.
>0.3	151 (31.0)	16 (48.5)	2.09 (1.03–4.26)	0.041		89 (22.1)	9 (23.7)	1.09 (0.50–2.39)	0.827	123 (27.0)		4 (23.5)	0.83 (0.27–2.60)	0.753
Age														
<65 years	186 (23.7)	7 (21.2)	Ref.			319 (79.4)	31 (81.6)	Ref.		213 (46.7)		11 (64.7)	Ref.	
≥65 years	301 (61.8)	26 (78.8)	2.29 (0.98–5.39)	0.057		83 (20.6)	7 (18.4)	0.87 (0.37–2.04)	0.745	243 (53.3)		6 (35.3)	0.48 (0.17–1.32)	0.153
Gender														
Male	299 (61.4)	23 (69.7)	Ref.			375 (93.3)	36 (94.7)	Ref.		319 (70.0)		14 (82.4)		Ref.
Female	188 (38.6)	10 (30.3)	0.69 (0.32–1.49)	0.344		27 (6.7)	2 (5.3)	0.77 (0.18–3.38)	0.731	137 (30.0)		3 (17.6)	0.50 (0.14–1.76)	0.281
Pathological stage														
I + II	237 (48.7)	11 (33.3)	Ref.			135 (33.6)	5 (13.2)	Ref.		353 (77.4)		10 (58.8)	Ref.	
III + IV	250 (51.3)	22 (66.7)	1.90 (0.90–4.00)	0.092		267 (66.4)	33 (86.8)	3.34 (1.27–8.74)	0.014	103 (22.6)		7 (41.2)	2.40 (0.89–6.46)	0.083
CCIS														
0	22 (4.5)	1 (3.0)	Ref.			343 (85.3)	27 (71.1)	Ref.		99 (21.7)		2 (11.8)	Ref.	
1–2	246 (50.5)	18 (54.5)	0.62 (0.79–4.88)	0.651		45 (11.2)	10 (26.3)	0.35 (0.16–0.78)	0.010	186 (40.8)		6 (35.3)	0.63 (0.12–3.16)	0.571
≥3	219 (45.0)	14 (42.4)	0.71 (0.89–5.67)	0.747		14 (3.5)	1 (2.6)	1.10 (0.14–8.70)	0.927	171 (37.5)		9 (52.9)	0.38 (0.08–1.81)	0.227
Length of stay, days, mean ± SD	25.1 ± 15.4	30.4 ± 23.1	1.01 (0.99–1.03)	0.082		23.8 ± 11.7	27.6 ± 13.3	1.02 (0.99–1.04)	0.079	23.2 ± 11.7		24.6 ± 13.2	1.01 (0.97–1.04)	0.627
Hemoglobin														
≥12 (g/dl)	140 (28.7)	8 (24.2)	Ref.			201 (50.0)	17 (44.7)	Ref.		175 (38.4)		7 (41.2)	Ref.	
<12 (g/dl)	347 (71.3)	25 (75.8)	1.26 (0.56–2.86)	0.580		201 (50.0)	21 (55.3)	1.24 (0.63–2.41)	0.536	281 (61.6)		10 (58.8)	0.89 (0.33–2.38)	0.816
Sodium														
≥135 (mEq/L)	396 (81.3)	25 (75.8)	Ref.			341 (84.8)	26 (68.4)	Ref.		392 (86.0)		13 (76.5)	Ref.	
<135 (mEq/L)	91 (18.7)	8 (24.2)	1.39 (0.61–3.19)	0.433		61 (15.2)	12 (31.6)	2.58 (1.24–5.39)	0.012	64 (14.0)		4 (23.5)	1.89 (0.60–5.96)	0.281
WBC														
<10,000 (/mm^3^)	302 (62.0)	19 (57.6)	Ref.			257 (63.9)	16 (42.1)	Ref.		259 (56.8)		11 (64.7)	Ref.	
≥10,000 (/mm^3^)	185 (38.0)	14 (42.4)	1.20 (0.59–2.46)	0.612		145 (36.1)	22 (57.9)	2.44 (1.24–4.79)	0.010	197 (43.2)		6 (35.3)	0.72 (0.26–1.97)	0.520

Abbreviations: CCIS, Charlson Comorbidity Index score; HNC, head and neck cancer; SD, standard deviation.

^a^ Holiday ratio: holiday/length of stay.

^b^ 30‐day PAR: 30‐day potentially avoidable readmission.

^c^ 95% CI: 95% confidence interval.

The multivariate logistic regression analysis revealed that the predictors of 30‐day PAR for those with colon cancer were a holiday ratio >0.3 (adjusted odds ratio [aOR] 2.16; 95% Confidence Interval [CI], 1.05–4.39) and age ≥65 years (aOR 2.36; 95% CI, 1.00–5.56). For those with HNC, P stage III + IV (aOR 3.58; 95% CI, 1.34–9.53), CCI 1–2 (aOR 3.36; 95% CI, 1.48–7.66), and WBC ≥10,000 (aOR 2.61; 95% CI, 1.30–5.22) were the predictors of 30‐day PAR (Table 3). There were no predictors of 30‐day PAR in those with other cancers.

**TABLE 3:** Multivariate regression for 30‐Day PAR^a^ by tumor sites

	Colon cancer		HNC		Other cancer (lung, liver, and prostate)	
Variables	aOR (95% CI^c^)	*p* value	aOR (95% CI)	*p* value	aOR (95% CI)	*p* value
Holiday ratio^b^ >0.3 (vs. Holiday ratio ≤0.3)	2.16 (1.05–4.39)	0.035			NA	
Age ≥65 (vs. Age <65 years)	2.36 (1.00–5.56)	0.050				
Pathological stage III + IV (vs. Pathological stage I + II)			3.58 (1.34–9.53)	0.011		
CCIS 1–2 (vs. CCIS 0)			3.36 (1.48–7.66)	0.004		
WBC ≥10,000 (vs. WBC <10,000)			2.61 (1.30–5.22)	0.007		

Abbreviations: aOR, adjusted odds ratio; CCIS: Charlson Comorbidity Index score; HNC, head and neck cancer; NA, not applicable.

^a^ 30‐day PAR: 30‐day potentially avoidable readmission.

^b^ Holiday ratio: holiday/length of stay.

^c^ 95% CI: 95% confidence interval.

**FIGURE 2:**
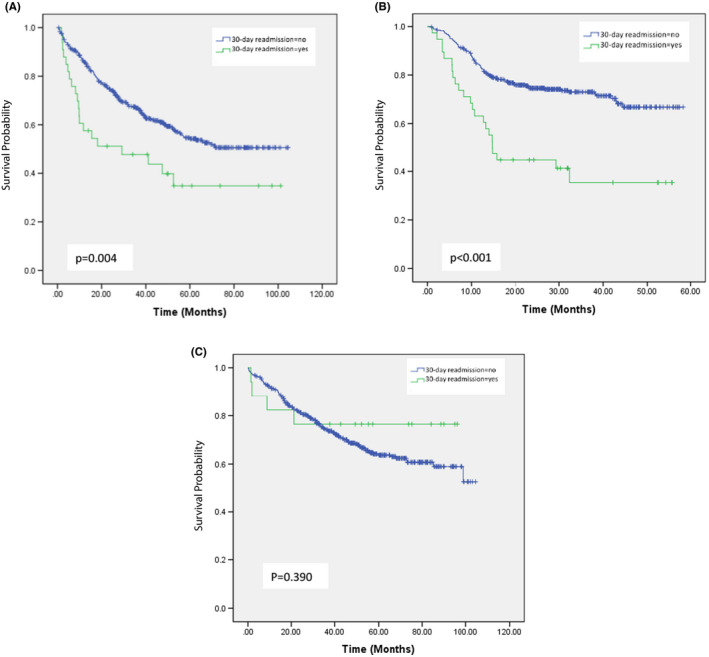
Kaplan–Meier survival curves for overall survival according to 30‐day readmission category for colon cancer (A), head and neck cancer (B), and other cancer(C) patients

Figure 2 showed the Kaplan–Meier survival curves according to 30‐day readmission category for colon cancer, HNC, and other cancer patients. The survival rates were statistically significantly higher in those patients without 30‐day readmission after discharge than in those with 30‐day readmission in the subgroups with colon cancer (*p *= 0.004) and HNC (*p *< 0.001).

## 4 DISCUSSION

Using a medical center cancer registry database and a clinical research database to examine the impact of continuity of care on 30‐day hospital readmissions for patients with cancer, a new proxy for continuity of care, the holiday ratio, was associated with 30‐day readmission. The holiday ratio >0.3 was a negative independent predictor of 30‐day PAR in patients with colon cancer. Even with the optimal care available in a medical center, the effect of the holiday ratio was seen in patients with colon cancer, which deserved our attention.

This study has several strengths. The data for our study were obtained from an inpatient database of our hospital which combined administrative and clinical database. So our analysis could control for these factors which could not be included in large nationwide database study. In our study, we developed a new proxy, the holiday ratio, which may better explain the continuity of care during hospitalization, and be simply applied to the worldwide hospital to predict potential higher risk patients with readmission during hospitalization.

Decreased continuity of care during hospitalization had been associated with poor outcomes or prolonged length of stay.^26^ Weekend usual provider continuity (UPC), which represented the fraction of the weekend days with a clinical note by a primary inpatient attending physician, has been used in internal medicine service.^14^ Higher weekend continuity of care was associated with reduced length of stay. Goodwin et al. proposed using a weighted mean of schedule continuity for hospitalists.^15^ A higher quartile of schedule continuity was associated with lower 30‐day readmission rates, lower 30‐day post‐discharge costs, and higher rates of discharge to the home. However, these above‐mentioned methods are not feasible in many hospitals. A new proxy for continuity of care during hospitalization was needed.

McAlister et al. found that the risk of readmission was significantly lower in patients discharged from teaching hospitals on weekend days. The authors attributed the better outcome to several factors uniquely present in teaching hospitals: the presence of more experienced physicians and nurses; standardized algorithms in place for disease management; better access to resources for patient education; and prompt outpatient follow‐up care.^6^ The samples in our study were from a medical center with higher quality of care. This quality may explain why a weekend discharge was not associated with readmission in our study.

Among the indicators for continuity of care, we explored a new proxy, the ratio of holiday days to the total length of stay, which may better explain the continuity of care during hospitalization and potentially be used to study heterogeneous diseases. At first, the whole cohort consisting of colon cancer, head and neck cancer, lung cancer, liver cancer, and prostate cancer was analyzed together. However, the impact of holiday ratio did not reach the significant level (Table S2). Due to heterogeneous tumor characteristics and caring styles, the analysis was performed separately. As shown by the result of our study, hospitalized colon cancer patients with a holiday ratio >0.3 had a higher risk of 30‐day readmission; there was no association for patients with HNC or other cancers. The different care styles of different staff could reduce the quality of care. A potential factor contributing to poor in‐hospital outcomes related to holidays may be handoffs of care, likely because of the increased number of team transitions during this time and the decreased compliance with the handoff structure itself.^27^ This greater exposure to handoffs may cause loss of information, either that communicated orally by patients and their families to a physician or that included in the electronic medical record. Reduced information flow may reduce patient trust and affect medical decision‐making and discharge planning.^26^

The long‐term impact of 30‐day readmission for patients with colon cancer, HNC, and other cancers was illustrated (Figure 2). The survival rates were statistically significantly higher in those patients without 30‐day readmission after discharge than in those with 30‐day readmission in the subgroups with colon cancer and HNC. This result is similar to that found by Hembree et al.^28^ Patients readmitted within 30 days of an unplanned hospitalization are at higher risk of mortality than those not readmitted. This result underscores the importance of reducing the occurrence of 30‐day readmission, an event which is related to the patient's subsequent long‐term treatment outcomes.

There are some limitations in our study. First, the data for our study were derived from an inpatient database, and errors in coding of diseases or procedures could exist. Our analysis is thus potentially subject to misclassification bias. Second, we could not control for other factors which may potentially affect readmission after hospital discharge, such as compliance with medications, dietary compliance, outpatient follow‐up, and discharge disposition. Third, the last known laboratory values such as hemoglobin and sodium levels were extracted from an electronic medical record database; the time between the examination date and the discharge date varied. Fourth, our cases are all malignant, and because of not enough events in our research, tumor location…et al. is not subdivided, and the related factors, education level … etc., are not included in the analysis, so as not to reduce the power of the research variables. Finally, the study used data from a single cancer center; external validation is therefore required in order to extrapolate results to other populations.

## 5 CONCLUSIONS

Among patients hospitalized in a medical center after cancer surgery, a higher holiday ratio during hospitalization in colon cancer patients affected the likelihood of subsequent 30‐day hospitalization. In order to provide better continuity of care throughout holiday times, strategies to improve the degree of cross‐over coverage are needed. Improving the hospitalist scheduling and appropriate coordination of care protocols on weekends during hospitalization can reduce the subsequent 30‐day readmission and mortality rates in colon cancer patients.

## CONFLICT OF INTEREST

The authors declare no relevant conflict of interest.

## AUTHOR CONTRIBUTIONS

**Ling‐Jan Chiou**: Study design, data analysis, data interpretation, and writing and review of the article. **Hsiu‐Min Chen:** data analysis and review of the article. **Li‐Fei Pan:** Study design, data analysis, and review of the article. **Ching‐Chih Lee:** Study design, data collection, data analysis, data interpretation, and writing and review of the article.

## ETHICS STATEMENT

Because we did not enroll any human participants or use personally identifiable records in our analysis, informed consent was not required. Our Ethics Committee of the Institutional Review Board approved the study protocol (VGHKS16‐CT‐3‐05).

## Supporting information

Supplementary Material

## Data Availability

Due to the IRB regulation in our institute, data are not available for public use at present.
