# Supplementary material for: Holiday ratio of hospitalization and 30‐day readmission rates among cancer patients after major surgery
Source: Cancer Med. 2021 Dec 14;11(3):743–52. doi: 10.1002/cam4.4482 (PMC8817097; doi:10.1002/cam4.4482)
Supplement: Supplementary file 1 — Supplementary Material [file CAM4-11-743-s001.docx]

|  | Date | 1 | 2 | 3 | 4 | 5 | 6 | 7 | 8 | 9 | 10 | 11 | 12 | 13 | 14 | 15 | 16 | 17 | 18 | 19 | 20 | 21 | 22 |
| --- | --- | --- | --- | --- | --- | --- | --- | --- | --- | --- | --- | --- | --- | --- | --- | --- | --- | --- | --- | --- | --- | --- | --- |
|  | Day | Fri | Sat | Sun | Mon | Tue | Wed | Thur | Fri | Sat | Sun | Mon | Tue | Wed | Thur | Fri | Sat | Sun | Mon | Tue | Wed | Thur | Fri |
|  | Holiday ratio |  |  |  |  |  |  |  |  |  |  |  |  |  |  |  |  |  |  |  |  |  |  |
| Pt 1 | 0.3125 |  |  |  |  |  |  |  |  |  |  |  |  |  |  |  |  |  |  |  |  |  |  |
| Pt 2 | 0.375 |  |  |  |  |  |  |  |  |  |  |  |  |  |  |  |  |  |  |  |  |  |  |
| Pt 3 | 0.3125 |  |  |  |  |  |  |  |  |  |  |  |  |  |  |  |  |  |  |  |  |  |  |
| Pt 4 | 0.25 |  |  |  |  |  |  |  |  |  |  |  |  |  |  |  |  |  |  |  |  |  |  |
| Pt 5 | 0.25 |  |  |  |  |  |  |  |  |  |  |  |  |  |  |  |  |  |  |  |  |  |  |
| Pt 6 | 0.25 |  |  |  |  |  |  |  |  |  |  |  |  |  |  |  |  |  |  |  |  |  |  |
| Pt 7 | 0.25 |  |  |  |  |  |  |  |  |  |  |  |  |  |  |  |  |  |  |  |  |  |  |

Descriptions: All patients had length of stay for 16 days. Patient 1-3 had a holiday ratio more than 0.3. Patient 4-7 had a holiday ratio less than 0.3.

**Supplemental Figure 1.** Diagram of Holiday ratio


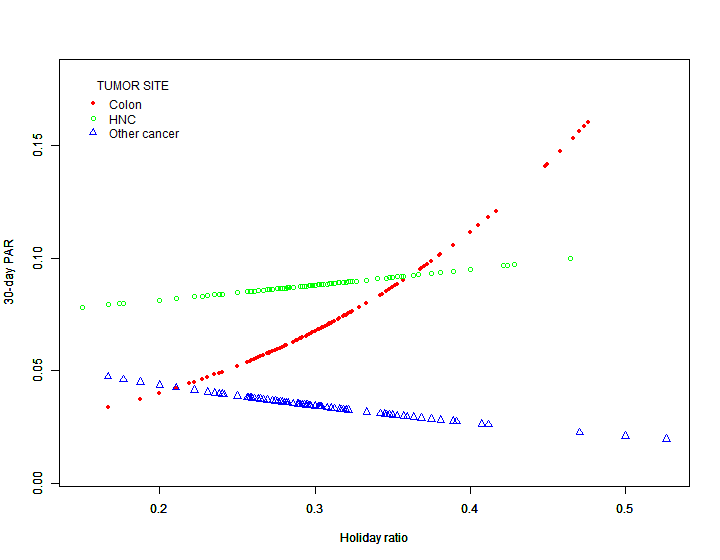


**Supplemental Figure 2.** Smooth curve fitting between holiday ratio and 30-day PAR.

We first use smooth curve fitting to examine whether the holiday ratio is partitioned into intervals for 30-day PAR used generalized additive model (GAM) to identify the non-linear relationship. Results appeared that the curves seemed have inflection point at approximately 0.3 in patients with colon cancer (p=0.097 < 0.10), but not in the other two groups (p=0.825 for HNC and p=0.656 for others) (Supplemental Figure 2).

We further performed piece-wise regression that is using a separate line segment to fit each interval. Log-likelihood ratio test was used to determine whether threshold exists to identify the inflection point. The results founded that inflection point were 0.3 in all patients or colon cancer group. Therefore, we adopted o.3 as the cut-off point to test the holiday effect. Additionally, we also performed stepwise logistic regression with Akaike information criterion and bootstrap method (1000 re-sampling) to robust regression estimators and the results were the same as in Table 3 (Supplemental Table 1). All of the analyses were performed with the statistical software packages R (http://www.R-project.org, The R Foundation).

**Supplemental Table 1**. Logistic Regression Models Predicting 30-Day PAR by tumor sites (Stepwise with AIC and bootstrap methods)

|  | Colon cancer | | HNC | | Other cancers  (Lung, Liver, prostate) | |
| --- | --- | --- | --- | --- | --- | --- |
| Variables | aOR  (95% CI) | P value | aOR  (95% CI) | P value | aOR  (95% CI) | P value |
| AIC:  Holiday ratio > 0.3  (vs. Holiday ratio ≤0.3) | 2.19  (1.07-4.49) | 0.032 | NA | NA | NA | NA |
| Bootstrap:  Holiday ratio > 0.3  (vs. Holiday ratio ≤0.3) | 2.54  (1.72-4.68) | --- | NA | NA | NA | NA |

AIC: Akaike information criterion.

aOR: Adjusted odds ratio; odds ratio were adjusted for age, P stage, CCI, and WBC.

**Supplemental Table 2**. Logistic Regression Models Predicting 30-Day PAR^a^ including tumor type for independent factors (full model)

| variables | aOR | 95% CI^b^ | | | P value |
| --- | --- | --- | --- | --- | --- |
| Tumor type (Colon cancer vs. Other cancer) | 1.25 | 0.66 | - | 2.36 | 0.489 |
| Tumor type (HNC vs. Other cancer) | 2.92 | 1.35 | - | 6.31 | 0.007 |
| Holiday ratio ^b^> 0.3 (vs. Holiday ratio ≤0.3) | 1.41 | 0.88 | - | 2.27 | 0.154 |
| Male( vs. female) | 1.39 | 0.76 | - | 2.56 | 0.286 |
| Age≥ 65(vs. Age < 65 yrs.) | 1.00 | 0.61 | - | 1.63 | 0.997 |
| Pathological stage III+IV (vs. Pathological stage I+II) | 2.32 | 1.40 | - | 3.85 | 0.001 |
| CCIS ^c^ 1-2 (vs. CCIS 0) | 2.37 | 1.19 | - | 4.73 | 0.045 |
| CCIS ^c^ ≥3 (vs. CCIS 0) | 2.27 | 1.02 | - | 5.06 | 0.881 |
| Hemoglobin ≥ 12( vs. Hemoglobin < 12 | 1.10 | 0.68 | - | 1.76 | 0.702 |
| Sodium ≥ 135 ( vs. Sodium < 135) | 1.68 | 1.01 | - | 2.79 | 0.044 |
| WBC ≥ 10000 (vs. WBC <10000) | 1.45 | 0.93 | - | 2.25 | 0.103 |

^a^ 30-day PAR: 30-day potentially avoidable readmission

aOR: adjusted odds ratio

^b^ 95% CI: 95% confidence interval
